# Management of a dentigerous cyst; a ten-year clinicopathological study

**DOI:** 10.1186/s12903-024-04607-w

**Published:** 2024-07-23

**Authors:** Afrah A. K. Aldelaimi, Hamid H. Enezei, Hibah Ezzat Rashid Berum, Suzan M. Abdulkaream, Khalil Abdullah Mohammed, Tahrir N. Aldelaimi

**Affiliations:** 1 Department of Oral Diagnosis, College of Dentistry, University Of Anbar, Ramadi, Iraq; 2 Department of Oral and Maxillofacial Surgery, College of Dentistry, University Of Anbar, Ramadi, Iraq; 3 Department of Oral Diagnosis, College of Dentistry, University Of Babylon, Babylon, Iraq; 4 Department of Histopathology, Ramadi Teaching Hospital, Anbar Health Directorate, Ramadi, Iraq

**Keywords:** Oral pathology, Histopathology, Oral cyst, Jaw, Mandible, Maxilla

## Abstract

**Background:**

Dentigerous cysts, deemed of developmental origin, are benign odontogenic cysts characterized by a gradual growth rate. Their occurrence is twice as prevalent in men compared to women. These cysts are recognized as the most frequent developmental cysts affecting the jaws, with a typical manifestation in individuals aged 20 to 40, while infrequently identified in young children. Notably, dentigerous cysts have the potential to attain significant dimensions, resulting in painless enlargement of the jaw and subsequent deformation.

**Objectives:**

To assess the clinicopathological features and management of ten years of experience with dentigerous cysts.

**Methods:**

A challenging cases were reported from reviewed records of the patients who were treated by the surgical intervention of various dentigerous cysts throughout the period of ten years, 2012–2022 and only histologically confirmed cases were selected, at Ramadi Teaching Hospital in addition to Rashid, Razi, Zuhur Private Hospitals and private clinics in Iraq.

**Results:**

76 patients were included in this clinicopathological research. The highest age group affected was ≤ 18 years (68.4%), 54% were male, the mandible was more affected (63.1%) than the maxilla (36.9%). Marsupialization was applied to 30.3% of the cases, while enucleation was carried out in 69.7%.

**Conclusions:**

The significance of meticulous examination of radiographs and the consequences associated with undetected and untreated ailments is affirmed by this case study. A comprehensive understanding of oral pathology serves as a valuable resource for dentists, facilitating accurate diagnosis, appropriate referrals, and the provision of anticipatory guidance to patients striving to achieve optimal oral health across various age groups.

## Introduction

Dentigerous cysts, is one of the oral cavity’s most prevalent developmental odontogenic cysts, prevalent among individuals aged 10 to 40 years, typically remain asymptomatic unless infected and are detectable solely through routine radiographic assessments. Originating from remnants of the reduced enamel epithelium encircling the crown of an unerupted or impacted tooth, these cysts become attached at the cementoenamel junction [1, 2]. Initially described by Paget in 1863 [3]. The term “dentigerous” signifies “containing tooth,” constituting approximately 15.2 to 33.7% of all odontogenic cysts [4].

The lesion most frequently affects the maxillary canine and mandibular third molar. Syndromes or systemic diseases, such as mucopolysaccharidosis and cleidocranial dysplasia, have been associated with unilateral and multiple cyst occurrences [5, 6]. Dentigerous cysts have the potential to induce asymmetries nerve alterations through compression, tooth displacement, and, in certain cases, malignant transformations into ameloblastoma, mucoepidermoid, or epidermoid carcinoma. Consequently, the choice of therapeutic intervention becomes pivotal. The reported incidence of dentigerous cysts stands at 1.44 for every 100 unerupted teeth, with a higher prevalence in males than females. Notably, these cysts are commonly linked to unerupted third molars, as well as first and second premolars and canines [8]. Thus, the current research aims to estimate the clinicopathological characteristics of dentigerous cysts within the patient population in Baghdad and Anbar provinces in, Iraq.

## Materials and methods

Encompassing the time frame from 2012 to 2022, this retrospective investigation included the reviewing of 92 patients that diagnosed with dentigerous cysts, only 76 cases have a complete recoreded data including histological confirmatiom. The research transpired at Ramadi Teaching Hospital, in addition to Razi, Zuhur, and Rashid Private Hospitals and private clinics in Baghdad and Anbar Provinces in Iraq. The clinical diagnosis of each case was reached after clinico-radiological evaluation and confirmed after the histological examination and then was included in this study. Comprehensive information pertaining to age, gender, disease type, cyst location, and clinical characteristics was extracted from each participant. Extraoral and intraoral clinical assessments were conducted, encompassing bony and soft tissues, employing meticulous inspection and gentle digital palpation to evaluate lesions thoroughly. Parameters such as consistency, size, fluctuation, shape, location, surface texture, color of overlying tissue, and examinations of locoregional lymphadenopathy were scrutinized. Various types of radiographic tests were employed, such as conventional radiography (in or out of the mouth), computed tomography (CT), and ultrasonic examination without or with FNAC (fine-needle aspiration cytology). The requisition of these radiological procedures and essential laboratory investigations was contingent upon each case’s clinical entity, medical history, and differential diagnosis.

**Fig. 1:**
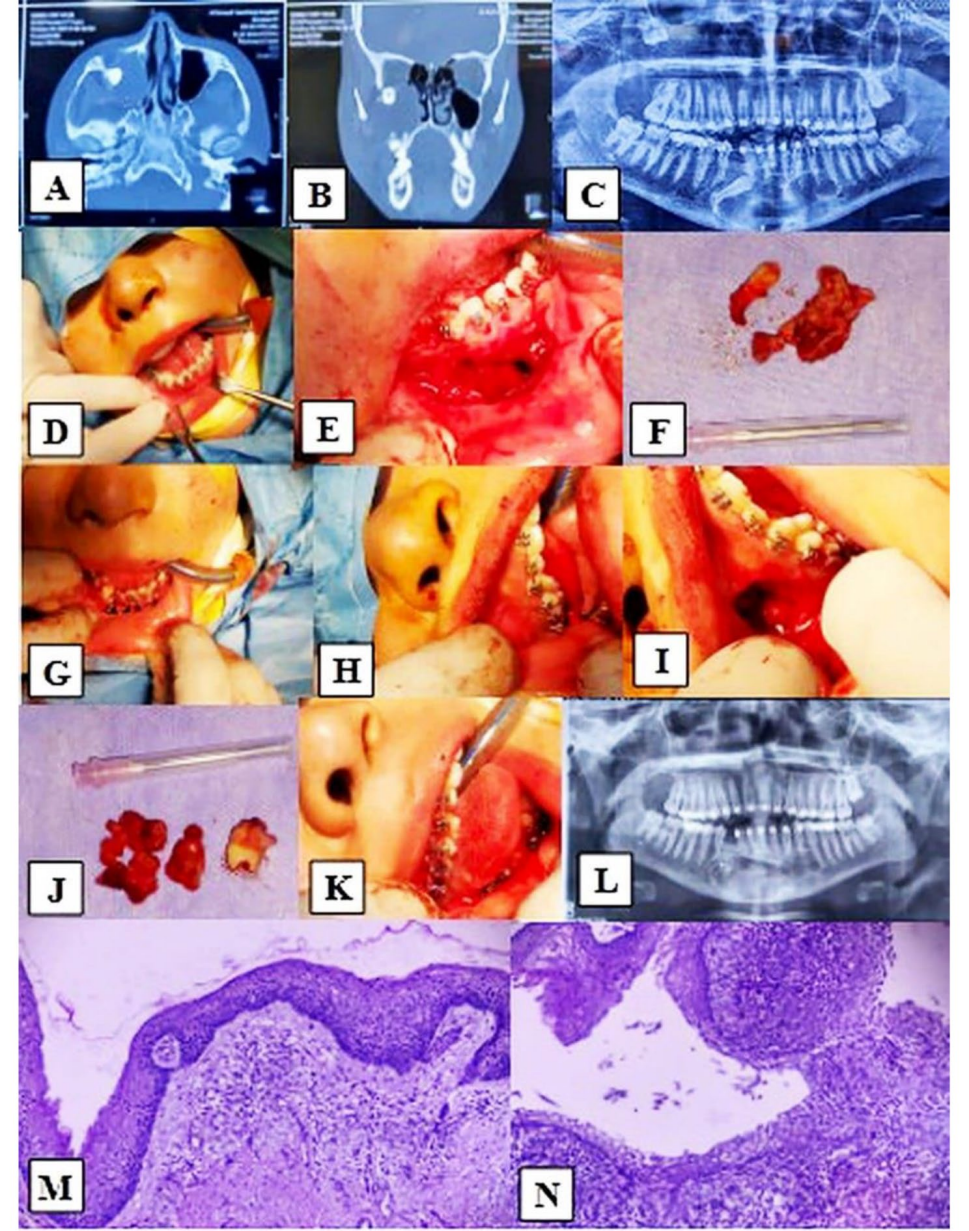
Radiological, surgical and histopathological findings of Dentigerous cysts in the maxilla and mandible. **A** and **B**: Axial and coronal CT scans confirmed dentigerous cysts, **C**: Preoperative OPG confirms dentigerous cysts for the mandible and maxilla sinus, **D**: Preoperative view for the mandible, **E**: Mandibular dentigerous cyst enucleation via a two-sided flap, **F**: Gross specimen of cystic lining with impacted lower right permanent canine, **G**: Suturing of flap, **H**: Preoperative view for the maxilla, **I**: Maxillary dentigerous cyst enucleation vial Caldwell-Luc approach, **J**: Gross specimen of cystic lining with an impacted upper ectopic right wisdom tooth, **K**: Suturing of flap, **L**: Postoperative OPG confirms the removal of the dentigerous cysts for the mandible and maxillary sinus, and **M** and **N**: Histopathological image demonstrates cystic lesion lined by nonkeratinized squamous epithelium

The surgical procedures were conducted with either local or general anesthesia, involving enucleation and marsupialization for cystic lesions, as well as the excision of jaws and facial bones. Various flap techniques were utilized in enucleation, including semilunar, two-sided or three-sided flaps, palatal envelope flaps, and the Caldwell-Luc operation. Mucoperiosteal reflection was carried out, and bone removal was executed either with a surgical handpiece or manually. The cyst membranes were exposed with meticulous care, subjected to curettage, and irrigated with normal saline. Subsequently, extraction or apicectomy of the impacted teeth or tooth was undertaken, followed by repositioning and suturing of the flap using a 3/0 black silk suture. In the case of marsupialization, the procedure involved exposing the superficial cyst lining, evacuating the contents of the cyst, suturing the lining to the adjacent mucosa, and packing the cystic cavity with iodoform gauze. Thorough irrigation with normal saline was administered, and patients received instructions to uphold optimal oral hygiene to prevent food debris accumulation and maintain the integrity of the surgical opening through subsequent follow-up visits. (Fig. 1).

After the surgical interventions, explicit postoperative guidelines were provided, and each patient was prescribed appropriate medications, including non-steroidal anti-inflammatory analgesics, antibiotics, and metronidazole. Intraoral antisepsis was maintained by employing Listerine mouthwash (Johnson & Johnson Ltd, UK) for 0.5 min during the postoperative time. Suture removal took place within 7–10 days postoperatively. Subsequently, at the Department of Histopathology, the entirety of the specimens underwent paraffin block embedding and hematoxylin and eosin staining for histopathological analysis. This examination involved scrutinizing the samples by two separate maxillofacial and oral pathologists and a general pathologist to confirm the diagnosis .

Additionally, sections of 5 μm thickness were affixed to positively charged microscopic slides to enhance tissue adherence for immunohistochemical staining (IHC). This staining utilized distinct monoclonal antibodies to accurately confirm the queried cases’ diagnostic status. All clinical and histopathological procedures adhered to ethical standards. It was sanctioned by the regional and institutional responsible human experimentation committee and adhered to the 2008 revised Helsinki Declaration in 1975. Ethical approvals for the study were obtained from the University of Anbar / Ethical Approval Committee; under Ref. No. 134 on 24/12/2023; also the Ethical Approval Committee was waived the need for informed consents for all patients. All data and materials used and analysed during the current study are available from the corresponding author.

All the data were statistically analyzed using Spss software version 26 as non-parametric data and tested by descriptive frequencies for each studied case and variable alone, followed by a ratio-statistics test for each separately.

## Results

Of the 92 individuals diagnosed with dentigerous cysts, 76 met the specified inclusion criteria and were included in the research. A total of 52 (68.4%) was the highest age group affected was ≤ 18 years, including 34 (44.7%) affecting the mandible, and 18 (23.7%) affecting the maxilla. There were 47 (61.8%) males and 29 (38.2%) females, with male predominance. These results are presented in Table 1.

Enucleation was applied in 53 (69.7%), including 32 (42.1%) for mandibular cases and 21(27.6%)for maxillary cases. In contrast, the Marsuplization was carried out in 23(30.3%) of the cases, including 16 (21%) and 7 (9.2%) for both jaws, respectively as demonstrated in Table 2.

Regarding the clinical presentations of the treated cases, 65 (85.5%) were presented as dentigerous cysts alone, including 38 (50%) for the mandible and 27 (35.5%) for the maxilla. Furthermore, 11 (14.5%) were present mixed with other anomalies like odontomas, including 10 (13.1%) for the mandible and 1 (1.3%) for the maxilla as shown in Table 3; since DCs associated with Odontomas are considered mixed radiolucencies depending on the stage of formation of odontoma and some of odontomas are associated with impacted unerupted teeth.

Concerning the radiographical findings, 59 (77.6%) were present as unilocular radiolucent lesions, and 17 (22.4%) were multilocular radiolucent lesions, as tabulated in Table 4.

Regarding surgical complications that present perioperatively or postoperatively, the occurrence of an oroantral fistula was 1 (1.3%), a nasal fistula was 1 (1.3%), nerve injuries were 3 (3.9%), excessive bleeding was 1 (1.3%), and a pathological fracture was found in 2 (2.6%) that was treated accordingly and no case was reprted recuurence.

**Table 1:** Demographic characteristics and the relationship between the clinical entity with age and gender in 76 patients with dentigerous cysts

Age group ( years)	*N* (%)	Site	
		Mandible *N* (%)	Maxilla *N* (%)
≤ 18	52(68.4%	34(44.7%)	18(23.7%)
> 18	24(31.6%)	14(18.4%)	10(13.2%)
Total	76(100%)	48(63.1%)	28(36.8%)
**Gender N (%)**			
Female	29(38.2%)		
Male	47(61.8%)		
Total	76(100%)		

**Table 2:** The relationship between the clinical entities with the surgical procedure in 76 patients with dentigerous cyst

Surgical Procedure	Site		
	Mandible *N* (%)	Maxilla *N* (%)	Total *N* (%)
Enucleation	32(42.1%)	21(27.6%)	53(69.7%)
Marsupialization	16(21%)	7(9.2%)	23(30.3%)
Total	48(63.1%)	28(36.8%)	76(100%)

**Table 3:** Clinical entities relationship with clinical presentation in 76 patients with dentigerous cyst

Clinical presentation	Site		
	Mandible *N* (%)	Maxilla *N* (%)	Total *N* (%)
Alone	38(50%)	27(35.5%)	65(85.5%)
Mixed ( associated)	10(13.1%)	1(1.3%)	11(14.5%)
Total	48(63.1%)	28(36.8%)	76(100%)

**Table 4:** The relationship between the clinical entities with the radiographical findings in 76 patients with dentigerous cyst

Radiographical findings	Site		
	Mandible *N* (%)	Maxilla *N* (%)	Total *N* (%)
Unilocular Radiolucency	34(44.7%)	25(32.9%)	59(77.6%)
Multilocular Radiolucency	14(18.4%)	3(3.9%)	17(22.4%)
Total	48(63.1%)	28(36.8%)	76(100%)

## Discussion

The dentigerous cyst is clinically characterized by tooth persistence, and painful bone swelling is absent with a firm consistency. Radiographically, two variations of dentigerous cysts have been identified: unilocular or multilocular radiolucent lesions surrounding the crown and extending along the root surface, giving the appearance that the entire tooth is enclosed within the cyst. Dentigerous cysts are authentic developmental cysts commonly linked with impacted teeth [6]. The primary diagnostic criterion is their cementoenamel junction attachment to the implicated teeth. Routine radiographic examination is typically instrumental in their identification. These cysts exhibit an association, in descending order of frequency, with maxillary canines, mandibular first premolar, mandibular third molars, maxillary third molars, mandibular canine, maxillary second premolar, and mandibular second premolars [7, 9, 10].

Radiographically, dentigerous cysts can manifest as distinct multilocular or unilocular radiolucencies encapsulating the unerupted tooth crown [11, 12]. Typically, the radiolucency originates at the tooth’s cementoenamel junction. In instances where a dentigerous cyst is linked with odontomas—lesions of odontogenic origin with an anonymous etiology or non-aggressive hamartomatous developmental malformations—it frequently remained asymptomatic. It went unnoticed till identified through routine radiography. The developmental origin of a dentigerous cyst is primarily linked to an unerupted tooth or, less commonly, to an odontoma. In cases of concurrent occurrence, the amalgamated lesion may attain a larger size, presenting an increased potential for substantial jaw destruction [13, 14].

Dentigerous cyst formation is considered to be due to fluid accumulation resulting in a cystic enlargement of the space between the reduced enamel epithelium of the follicle and the enamel of the crown [9]. Initiating factor for at least some dentigerous cysts could be periapical inflammation arising from an overlying primary tooth in close proximity to the follicle [8, 10].

Microscopic analysis of the decalcified hard tissue sections revealed eosinophilic material with a dentin-like structure. The soft tissue displayed a thin, nonkeratinized epithelial cystic lining characteristic of a dentigerous cyst. The definitive diagnosis was established as a “dentigerous cyst from a complex odontoma.” The dentigerous cyst’s histopathological features included a pathological cavity flanked by intense chronic inflammation and hyperplastic epithelium. Additionally, dentigerous cyst characteristics consisted of a thin fibrous cystic wall sounded by two to three thick layers of stratified non-keratinizing squamous epithelium, along with scarce inflammatory infiltration in the cellular connective tissue [15, 16]. Potential differential diagnoses for such radiolucency encompass radicular cysts, odontogenic keratocysts, and odontogenic tumors such as ameloblastoma, Pindborg tumors, odontomas, and cementomas [9, 10].

The most prevalent associated complications encompass carcinomas or neoplastic alterations, potentially giving rise to epidermoid carcinoma emerging from the epithelial lining of the cyst or squamous cell carcinoma. Another complication involves the fracture of the pathologic jaw, which may transpire when the cyst is entirely eroded, particularly in the infrequently encountered posterior bone, resulting in a secondary infection or pathologic bone fracture. Secondary infections can ensue, resulting in additional complications [11]. Enucleation, coupled with the correlated supernumerary tooth extraction, represents the dentigerous cyst standard treatment [12–14]. General dental practitioners frequently assume a pivotal role in suggesting exposure to relevant radiographs and recording outcomes for the dentist’s subsequent evaluation. This instance serves as a method to enhance dental hygienists’ awareness of the potential manifestation of dentigerous cysts in toddlers, deviating from the more typical presentation around maxillary canines and third molars. The potential for neoplastic transformation and infiltration beyond the cyst wall substantiates the comprehensive enucleation of the dentigerous cyst, emphasizing the importance of histopathological examination [15–18].

Enucleation of the cyst is considered the treatment of choice whenever possible. However, due to the degree of destruction generated by this technique, other more conservative options such as marsupialization or endoscopy, cryotherapy, exodontia or a combination of treatments designed to preserve adjacent anatomical structures are recommended. The technique used varies depending on the damage and patient characteristics. Therefore, all variables must be well evaluated before choosing a treatment to ensure patient safety and comfort [3, 9, 10]. Marsupialization is advocated for extensive cysts when a solitary drain might prove ineffective, and the full elimination of the encompassing structure is not preferable for a cyst of considerable size. Conversely, alternative research suggests enucleation with or without prompt grafting of bone. In the current instance, the impacted premolar and enucleation surgical extraction was conducted without bone grafting for the associated cyst [19–22].

## Conclusion

It is advised that choosing a treatment technique for a dentigerous cyst might not be arbitrary but rather appropriate to the distinctive needs of patients. This customization should take into account factors such as the cyst’s location and size, the age of the patient, the impacted dentition, root completion status in the associated tooth, connection to the surrounding structures, the clinical courses, histological presentation, and the patient adherence to a prescribed course of care. The current study provides increased knowledge of the histological features of dentigerous cysts and provides further evidence regarding the frequency of inflammatory dentigerous cysts. The concurrent presence of these pathologies is infrequent, and diagnosing them based solely on radiographic appearance presents a challenging task.

## Data Availability

All data and materials used and analysed during the current study are available from the corresponding author.
